# A metagenomic approach from aphid’s hemolymph sheds light on the potential roles of co-existing endosymbionts

**DOI:** 10.1186/s40168-015-0130-5

**Published:** 2015-12-15

**Authors:** Caroline De Clerck, Akiko Fujiwara, Pauline Joncour, Simon Léonard, Marie-Line Félix, Frédéric Francis, M. Haissam Jijakli, Tsutomu Tsuchida, Sébastien Massart

**Affiliations:** Urban and Integrated Plant Pathology Laboratory, Gembloux Agro-bio Tech, University of Liège, 2 Passage des Déportés, 5030 Gembloux, Belgium; Functional and Evolutionary Entomology Laboratory, Gembloux Agro-bio Tech, University of Liège, 2 Passage des Déportés, 5030 Gembloux, Belgium; Graduate School of Science and Engineering, University of Toyama, 3190 Gofuku, Toyama, Toyama, Japan; Chemical Genomics Research Group, RIKEN Center for Sustainable Resource Science, 2-1 Hirosawa, Wako, Saitama, Japan

**Keywords:** *Pentalonia nigronervosa*, *Wolbachia*, Co-obligatory symbiosis

## Abstract

**Background:**

Aphids are known to live in symbiosis with specific bacteria, called endosymbionts which can be classified as obligate or accessory. *Buchnera aphidicola* is generally the only obligatory symbiont present in aphids, supplying essential nutrients that are missing in the plants phloem to its host. *Pentalonia nigronervosa* is the main vector of the banana bunchy top virus, one of the most damageable viruses in banana. This aphid is carrying two symbionts: *B. aphidicola* (BPn) and *Wolbachia* sp. (wPn). The high occurrence of *Wolbachia* in the banana aphid raises questions about the role it plays in this insect. The goal of this study was to go further in the understanding of the role played by the two symbionts in *P. nigronervosa.* To do so, microinjection tests were made to see the effect of wPn elimination on the host, and then, high-throughput sequencing of the haemolymph was used to analyze the gene content of the symbionts.

**Results:**

We observed that the elimination of wPn systematically led to the death of aphids, suggesting that the bacterium could play a mutualistic role. In addition, we identify and annotate 587 and 250 genes for wPn and BPn, respectively, through high-throughput sequencing. Analysis of these genes suggests that the two bacteria are working together for the production of several essential nutrients. The most striking cases are for lysin and riboflavin which are usually provided by *B. aphidicola* alone to the host. In the banana aphid, the genes involved in the production pathways of these metabolites are shared between the two bacteria making them both essential for the survival of the aphid host.

**Conclusions:**

Our results suggest that a co-obligatory symbiosis between *B. aphidicola* and *Wolbachia* occurs in the banana aphid, the two bacteria acting together to supply essential nutrients to the host. This is, to our knowledge, the first time *Wolbachia* is reported to play an essential role in aphids.

**Electronic supplementary material:**

The online version of this article (doi:10.1186/s40168-015-0130-5) contains supplementary material, which is available to authorized users.

## Background

Bananas and plantains, belonging to *Musa* sp. genus are among the 10 most important staple food worldwide with a global production of 13.9 million tons in 2012 (FAO stat, 2014). They are important for food security, feeding millions of small growers, and as a cash crop in many developing countries.

*Pentalonia nigronervosa* Coquerel, the banana black aphid is the main vector of one of the most damageable viruses in banana, causing important production losses: the banana bunchy top disease (BBTD) [1]. Despite its negative impact on banana production, little is known about this aphid species and its bacterial symbionts. A recent study [2] showed that all tested *P. nigronervosa* individuals carried two endosymbionts: *Buchnera aphidicola* (BPn) and *Wolbachia* sp*.* (wPn). The systematic presence of *Wolbachia* in an aphid species is quite rare and its high prevalence (100 %) in the banana aphid suggests that it could play some important roles in *P. nigronervosa*.

Symbiosis between bacteria and insects is a well-known phenomenon [3, 4]. Aphids in particular, harbor several bacterial endosymbionts that range from obligate to accessory [5]. Obligate or primary symbionts are vital for aphid’s survival, as they supply to their hosts essential nutrients that are missing in the plants phloem [6]. Many aphid species also harbor facultative or secondary symbionts which can differ not only among host species but also among individuals within a single species [7]. Those are not required for the survival of their hosts but can have several effects, either positive or negative: protection of the host against high temperatures or natural enemies like parasitoids or fungi [8], loss of fecundity, cytoplasmic incompatibility [9], negative fitness effect [10] or effect on host plant specialization [11].

*Buchnera aphidicola* is an obligate symbiont present in almost all aphid species [12, 13]. This gamma-protebacterium is located in the cytoplasm of hypertrophied specialized cells of the aphid’s body called bacteriocytes [14]. There is a strong link between *Buchnera* and its host, each partner of the symbiosis being incapable of living without the other [15]. *Buchnera* provides to its host essential amino acids that are absent in plant phloem. In exchange, the aphid provides a stable niche and nutrients for the bacterium [6, 13].

*Wolbachia* sp. are intracellular bacteria widely detected at relatively high frequency from diverse insects, other arthropods, and nematodes (with a current prevalence of at least 65 % in arthropod species [16]). Nevertheless, its presence in aphids is rarer. *Wolbachia* has indeed only been detected in 22 aphid species so far, among the 4000 species that have been described until today [17, 16].

*Wolbachia* often manipulate the host reproduction, by inducing parthenogenesis, cytoplasmic incompatibility, male killing, and feminization [18–21]. Moreover, some positive and mutualistic roles of this bacterium have been discovered recently in insects: it was shown to play a nutritional role not only in *Drosophila melanogaster* but also in the bedbug *Cimex lectularius*, and it is believed to be able to manipulate the host antioxidant system in a manner that is beneficial to host survival [22, 23]. It was also reported that this bacterium could provide protection against viral infections to their hosts, especially in the case of *Drosophila melanogaster* [24].

In this paper, we show that the two symbionts, *B. aphidicola* and *Wolbachia* sp., co-localize in the bacteriocytes of *P. nigronervosa*. We also observed that the selective elimination of wPn led to the death of aphids, suggesting that the bacterium could play a mutualistic role for the host. In addition, genome analyses of the symbionts allow us to discover that the two bacteria seem to work in association and share metabolites to produce all the nutrients needed by their host to survive on its phloem diet.

This is, to our knowledge, the first report suggesting a potential mutualistic association between *Wolbachia* and an aphid species. By increasing the knowledge about the relations existing between the aphid and the bacteria, the data provided in this paper could be a first step in the development of a sustainable way to control the banana aphid and thus the spread of BBTD infection.

## Results

### Confirmation of symbiotic content of *P. nigronervosa* and comparison between strains

PCR analysis targeting bacterial 16S ribosomal RNA genes showed that all the individuals tested for each strain carried both *Wolbachia* and *B. aphidicola*. The symbionts were always present whatever the origin of the aphid strain (Africa, South America, or Australia). This result is consistent with the ones obtained in a precedent study [2], where 20 individuals of 6 banana aphid strains coming from different geographic origins were found to carry both symbionts. Moreover, the multilocus sequence typing (MLST) gene alignment for *Wolbachia* showed that all the compared strains of wPn were 100 % identical.

### *In vivo* localization of the symbionts

**Fig. 1:**
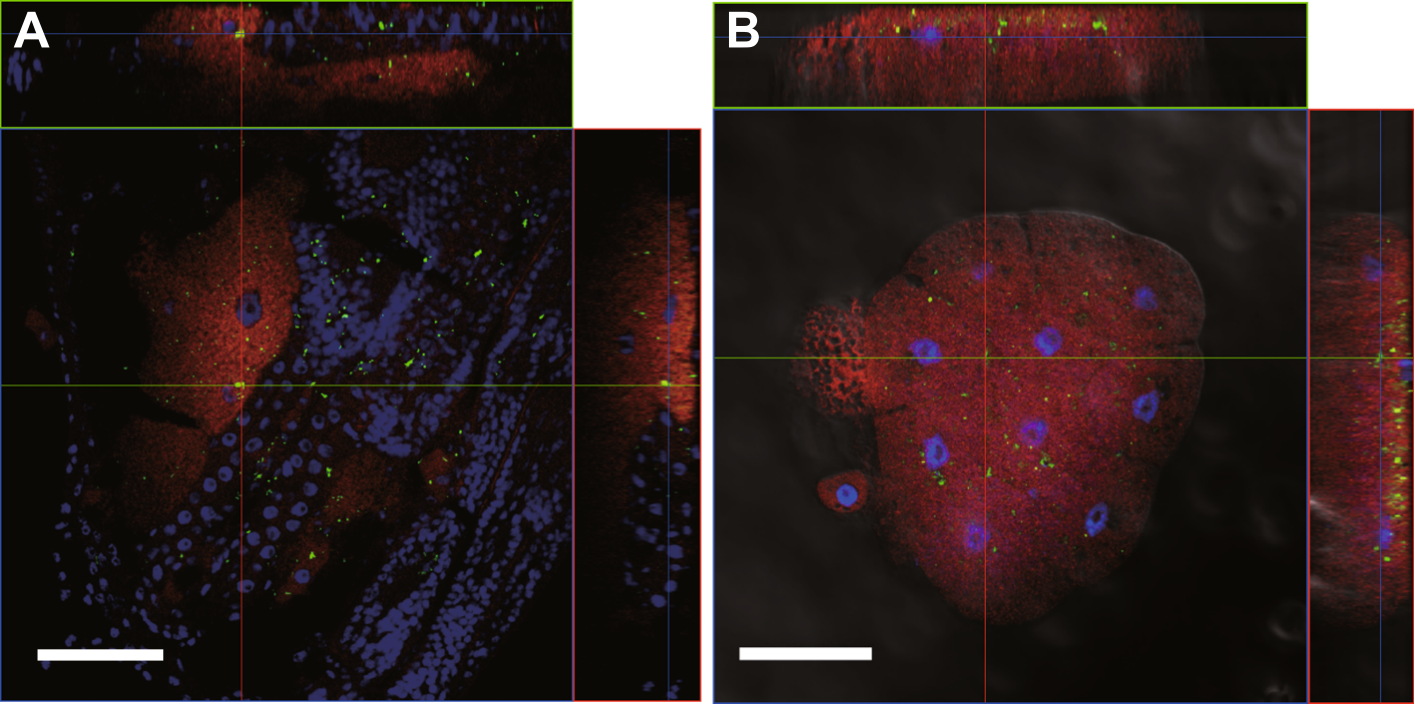
In situ hybridization of Bpn (in *red*) and wPn (in *green*) in *P. nigronervosa*. **a** Aphid embryo and **b** bacteriocytes dissected from the adult aphid. The z-stack orthogonal view confirms the signals of *Wolbachia* exist within the bacteriocytes. *Bars*, 50 μm

Whole-mount fluorescence *in situ* hybridization of dissected embryos revealed *in vivo* localization of *Buchnera* and *Wolbachia. Buchnera* was found in bacteriocytes, while *Wolbachia* was detected in various aphid tissues including bacteriocytes (Fig. 1a). In adult aphids, *Wolbachia* also co-existed with *Buchnera* in the bacteriocytes (Fig. 1b). No-probe control and ribonuclease (RNase)-digested control did not show any signals of the two symbionts.

### Selective elimination of *Wolbachia*

On 90 G0 aphids injected, all gave birth to several G1 nymphs. For Cefotaxime treatments, G2 and G3 nymphs were also produced. Ten adults of each generation were tested by PCR and all were shown to carry both *B. aphidicola* and *Wolbachia*. For tetracycline treatments, all the G1 nymphs died before reaching the adult stage. Twenty dead nymphs were collected and tested, the tests showing the presence of *Buchnera* but not of *Wolbachia*. The 30 G0 adults were also tested and all carried the two symbionts.

Tetracycline has been reported to have negative effect on *Buchnera* as well as on *Wolbachia*. The obtained results can here be explained by the concentration of *Wolbachia* in the bacteriocytes, which seems to be much smaller than the one of *Buchnera*. The antibiotic treatment thus eliminated *Wolbachia*, but only decreased *Buchnera* quantity, without eliminating it completely. The observed aphid’s mortality therefore seems to come only from *Wolbachia* elimination.

Ampicillin treatments generated similar results. Here, some of the G1 nymphs reached the adult stage and gave birth to viable G2 and G3 generations, but most of the G1 nymphs died before reaching the adult stage. Twenty dead nymphs were collected and tested, showing only the presence of *Buchnera*. All the G0 adults and four adults that reached the G3 have been tested, showing the presence of both *Buchnera* and *Wolbachia*. All the aphids injected with water stayed alive and gave birth to three generations of aphids.

### Sequencing and contig assemblies

The sequencing run generated 33.4, 37.8, and 33.6 million of high quality sequences of 2 × 100 nt for Madagascar, Burundi, and Gabon strains, respectively. All the pooled reads were assembled into 243,961 contigs with a total length of 99.8 Mb, corresponding to an average size of 409 nt (N50 = 421 nt). The contigs were annotated by blastx, and the contigs matching with endosymbionts sequences were selected. The genome of BPn was covered by 587 contigs, representing 579 kb with an average contig size of 986 nt and an average coverage of 858X. The G + C content was 27 %. The genome of wPn was covered by 1409 contigs, representing 1312 kb (average size of 931 nt) with an average coverage of 1041X. The G + C content was 33 %.

The cumulated size of contigs from BPn and wPn genomes, e.g., 579 and 1312 kb respectively, is very close to the size of the published complete genome sequences in the databases (see Additional files 3 and 4). Similarities in the GC percentages have also been observed.

### Gene contents of endosymbionts and comparative genomics

Analysis of the 16S ribosomal ribonucleic acid (rRNA) gene sequences generated after high-throughput sequencing confirmed the sole presence of *B. aphidicola* and *Wolbachia* sp. in the banana aphid (see Additional file 2).

**Fig. 2:**
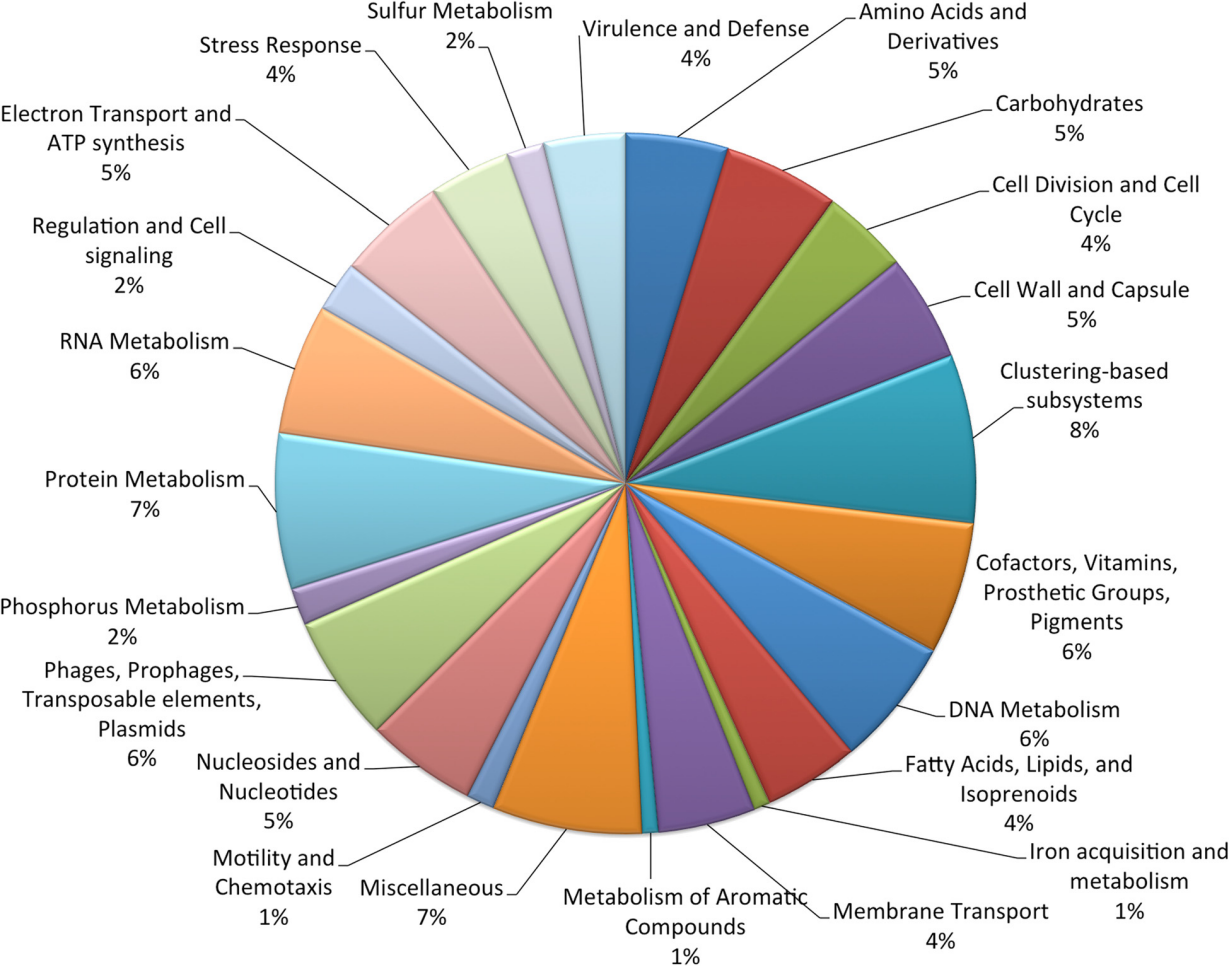
Distribution into functional roles categories of the 587 genes encoded in the 1312-kb partial sequence of the wPn genome

In addition, our study allowed us to identify and annotate 587 genes for wPn. Figure 2 shows the distribution of those genes into functional role categories. We can see that 5 and 6 % of those genes are related to amino acid or vitamin metabolism, respectively, which is slightly above what was observed for wBm and wMel [25]. In addition, 1 % of the genes are related to iron acquisition and metabolisms and 6 % to prophages and transposable elements, which are common features in *Wolbachia*’s genomes [25–27]

Among those 587 genes, 427 were common with the 6 *Wolbachia*’s reference genomes (see Additional file 3) currently available in Genebank and 84 in common with 5 of them. In addition, we identified 5 genes in wPn that were not present in the 6 reference genomes, with two of particular interest. The first one was a gene coding for an acetyl-CoA acetyltransferase (EC 2.3.1.9), which is an enzyme that catalyzes the reaction: 2 acetyl-CoA⇌CoA + acetoacetyl-CoA. This is interesting as most symbionts lacks CoA, which has to be supplied by the host [25]. The substrate of the reaction, acetyl-CoA, could be produced through the pyruvate decarboxylation complex, from which we have sequenced two of the three implicated enzymes in wPn.

The second was a gene coding for aminoglycoside phosphotransferase, an enzyme conferring resistance to many aminoglycoside antibiotics by regiospecific phosphorylation of their hydroxyl groups [28].

**Fig. 3:**
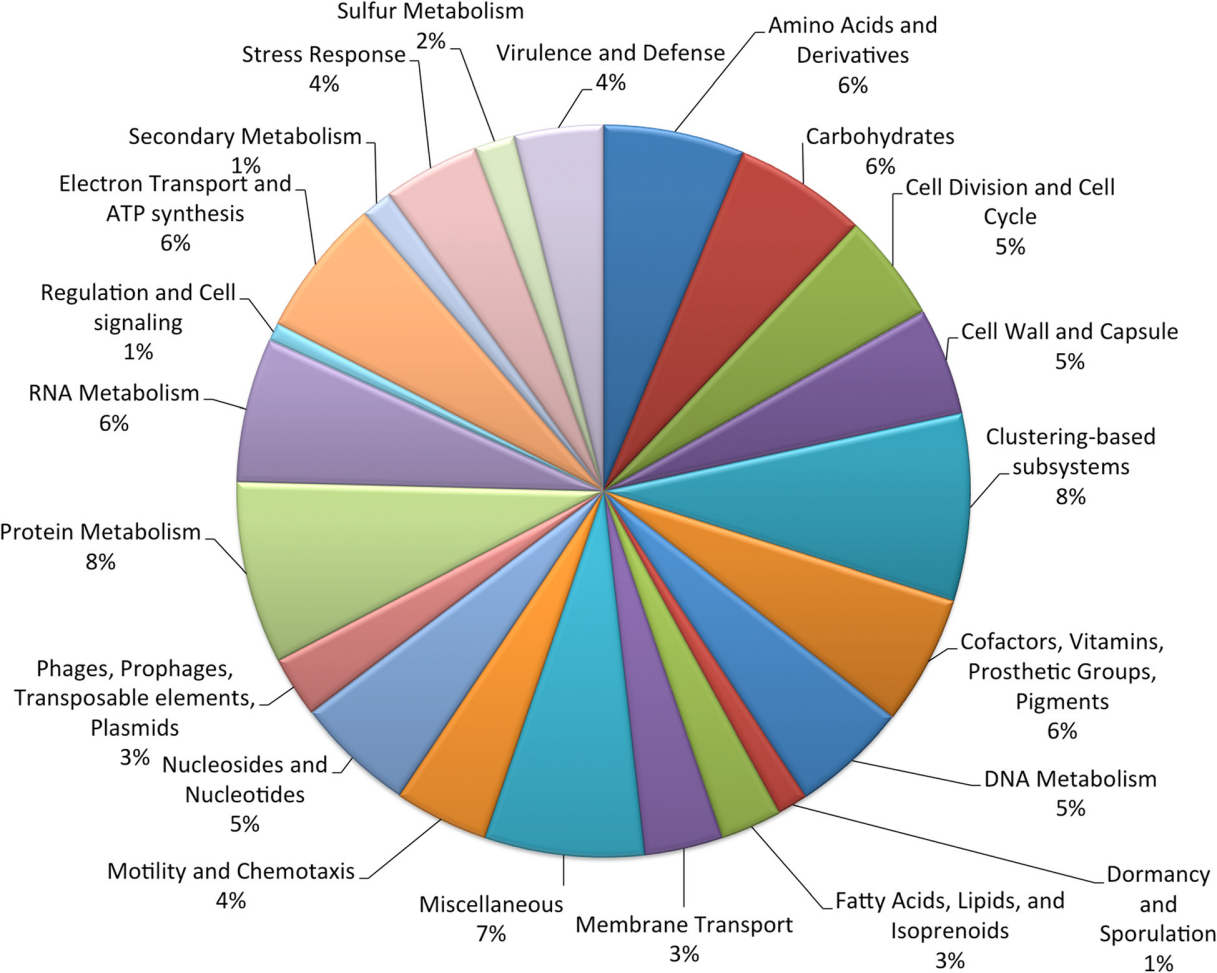
Distribution into functional roles categories of the 250 genes encoded in the 579-kb partial sequence of the BPn genome

Two hundred and fifty genes were annotated for *Buchnera*, among which 82 were in common with the 7 reference genomes available in Genebank (see Additional file 4). Three genes were identified to be unique to *Buchnera* of *P. nigronervosa*. Figure 3 shows the distribution of those 250 genes into functional role categories.

**Fig. 4:**
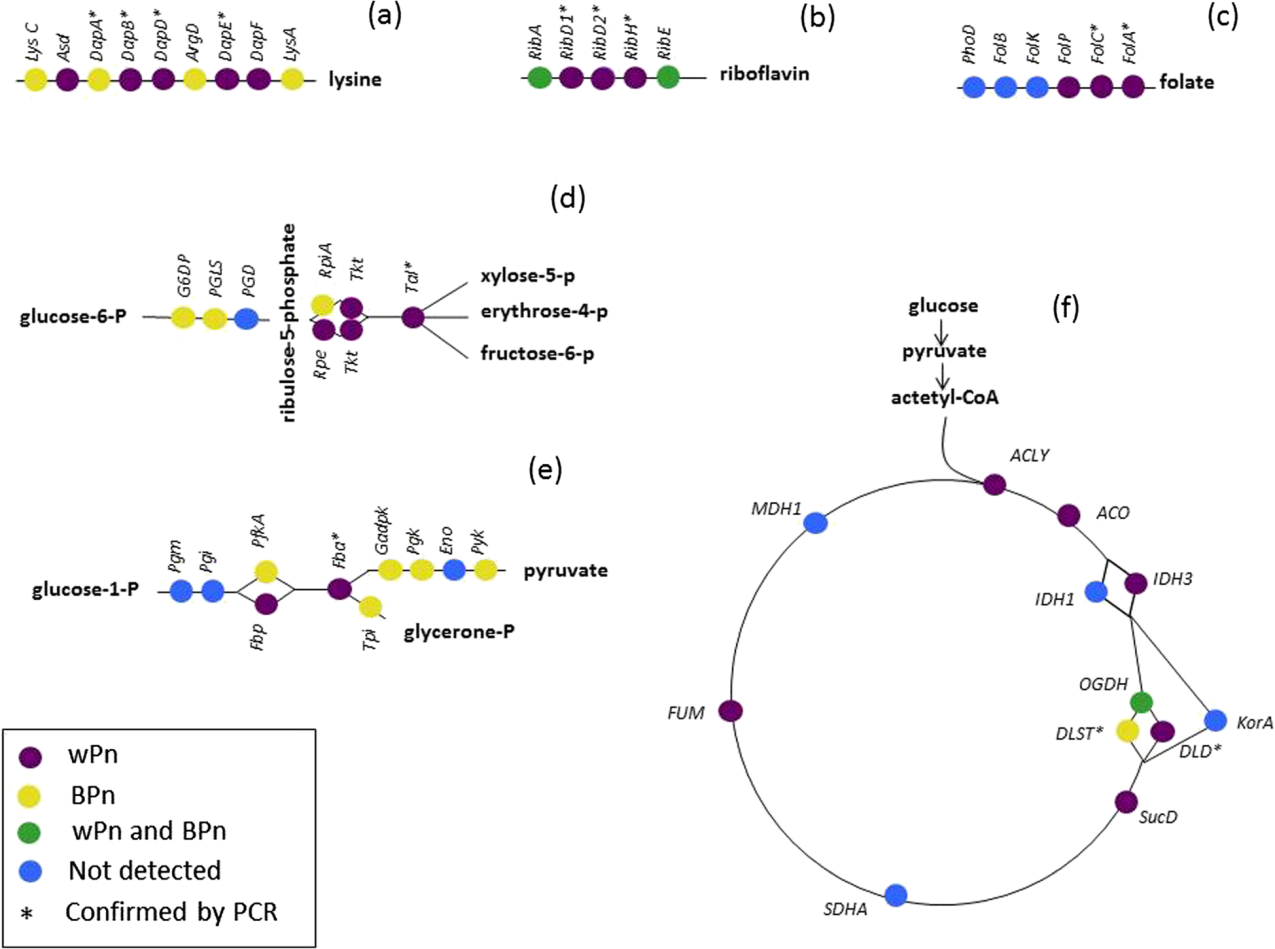
Pathways reconstruction showing repartition of genes that were detected in BPn (in *yellow*), in wPn (in *purple*), or in both symbionts (in *green*). Genes marked with a star were confirmed by PCR. **a** lysin pathway, **b** riboflavin pathway, **c** folate pathway, **d** pentoses phosphates pathway, **e** glycolysis pathway, and **f** citrate cycle pathway

Among the annotated genes from the two endosymbionts, some were of particular interest because of their implication in energetic, amino acids or vitamin production pathways. Figure 4a–f shows that wPn (in purple) and BPn (in yellow) complement each other in several pathways. The most striking cases can be found for lysine (Fig. 4a) and riboflavin (Fig. 4b) production, which are nutrients essential for the host and not known to be present in their diet.

### Confirmation experiments

The presence of the genes in the highlighted pathways was confirmed independently in the endosymbionts of the three tested aphid strains by reads mapping of each sample individually vs. the de novo assembled contigs.

Moreover, as the complete genomes for BPn and wPn were not available, the observations made with NGS about the absence or presence of genes coding for essential enzymes in each pathway were confirmed by PCR using degenerated primers for both bacteria.

Even if it is generally difficult to prove the absence of a gene, the highly reduced and conserved genome of *Buchnera* allowed us to design primers targeting conserved regions in the genes tested, based on all the published *Buchnera* sequences available in databases. The validity of the primers was tested using *B. aphidicola* of *A. pisum* (BAp) as a positive control. In the same way, primers were designed to amplify genes of *Wolbachia* using the same method and all the *Wolbachia* genomes available in databases.

The targeted genes are marked with a star in Fig. 4a–f. The PCR tests seem to indicate the absence, in BPn, of 11 essential genes that were only detected in wPn by Illumina sequencing. These genes were confirmed to be present in BAp.

In addition, three genes that were identified only for BPn were not detected in wPn after PCR amplifications. The later genes were amplified in BAp.

## Discussion

### Symbiotic content of *P. nigronervosa*

The detection of *B. aphidicola* and *Wolbachia* sp. as unique symbionts by high-throughput sequencing is consistent with the data generated in a precedent study [2] and seems to validate our methodology of collection, as no 16S rRNA gene coming from the aphid’s gut bacteria was recovered. Moreover, the analysis of samples coming from various geographical areas confirmed the presence of both endosymbionts.

### *Wolbachia* elimination and localization

We observed that all the antibiotics that eliminated wPn have led to the death of the aphid host. It suggests then that the presence of wPn is vital for the banana aphid, as it is the case for BPn. This observation, in addition to the fact that the two bacteria are localized close to each other within the bacteriocytes, sharing the same ecological niches within the host body (which must facilitate various biological interactions between the co-existing symbionts) let us hypothesize that a mutualistic relationship could exist between wPn and the aphid.

Bacteriome-associate symbionts of sap-feeding insects are usually mutually obligate with their hosts, being completely dependent of each other [29]. Studying the genome of the two co-resident symbiont allowed us to go further into the investigation of this possible tripartite mutualism.

### Pathways analysis

Metabolism’s predictions based on the genome sequence are particularly important in the case of uncultivable organisms [3]. Our metabolic reconstructions of BPn and wPn genomes seem to indicate that the two bacteria are working together for the production of certain essential nutrients for their host.

1. Amino acid synthesis

*Buchnera aphidicola*, the obligatory symbiont present in nearly all aphid species, is known to provide essential amino acids to its host. It has then retained all the genes needed for their biosynthesis [30].

As expected, BPn is able to synthetize almost all those essential amino acids. However, some pathways seem to be incomplete, with some genes absent. This situation could have been very problematic for the host in the absence of wPn, but the latter bacterium presents a perfect complementarity with BPn in several pathways.

The case of the lysine production pathway is noteworthy (Fig. 4a), with a pathway shared between BPn and wPn, with none of the bacterium capable of synthetizing lysine alone. Lysine is an essential amino acid that the aphid host cannot synthetize or find in its diet and which is usually provided by *B. aphidicola* to the host [31].

Moreover, our data show that BPn lacks the precursor erythrose phosphate, needed for the biosynthesis of tryptophan, tyrosine, and phenylalanine amino acids. This precursor seems to be supplied by wPn through the non-oxidative part of pentose phosphate pathway. Here again, the complementarity of the metabolic pathways suggests a close collaboration of both endosymbionts to supply essential nutrients to their host.

2. Vitamins and cofactors

wPn possesses the entire pathway of folate (Vitamin B9) production (Fig. 4c), while it is absent in BPn, as in other *Buchnera* species [32]. Folate is essential in insects and aphids development [33], but is believed to be found in plant phloem [34].

More interestingly, the pathway for riboflavin (B2 vitamin) production seems to be shared between the two symbionts (Fig. 4b). Riboflavin has been shown to be essential for insects and aphids [35] and is usually synthetized by *Buchnera* only [36], the pathway being missing in aphid’s genomes [37]. In addition, this vitamin is absent in plant phloem [38]. The provision of this vitamin by a *Serratia* symbiont to aphid is at the basis of a co-obligate symbiosis in *Cinara tujafilina* [37].

3. Pentose phosphate pathway

BPn possesses all the genes needed to go through the oxidative part of the pathway, responsible for the production of NADPH, implicated in many reducing processes and in fatty acid and nucleotides synthesis. All the genes coding for the non-oxidative part of the pathway are missing in BPn but present in wPn. In particular, wPn has the enzymes necessary for the supply of erythrose 4 phosphate, which is an important precursor in phenylalanine, tyrosine, and tryptophan biosynthesis, which are essential amino acids for the aphid (Fig. 4d).

4. Glycolysis/gluconeogenesis

As it was already observed for other *B. aphidicola* strains [39], the glycolysis pathway, in which glucose is converted in pyruvate with energy production, seems to be used by BPn for sugar utilization. 6-phosphofructokinase (EC 2.7.1.11), the key enzyme of glycolysis is indeed present. No hexokinase have either been detected, suggesting that the bacterium has to rely on its host for the supply of hexoses. BPn also lacks the key genes implicated in neoglucogenesis.

In wPn, like in *Wigglesworthia* [39], the genome lacks the gene to encode phosphofructokinase but contains the fructose biphosphatase (EC 3.1.3.11), the key enzyme of the gluconeogenesis. In this pathway, glucose is produced from non-glucidic precursors such as pyruvate or amino acids. wPn, as it was also observed in wMel [26], seems to be able to oxidize hexoses to pyruvate using the non-oxidative branch of the pentose phosphate pathway, which is complete in that organism.

It is therefore tempting to consider the possibility of complementarity of the two symbionts: BPn supplying pyruvate to wPn, the latter supplying glucose to BPn (Fig. 4e).

The two endosymbionts possess the genes coding for the pyruvate dehydrogenase complex leading to the production of acetyl-CoA by oxidation of the pyruvate in the presence of CoA. WPn seems however to be the only one able to synthetize CoA through a gene coding for an acetyl-CoA acetyltransferase (EC 2.3.1.9), catalyzing the conversion of acetyl-CoA into CoA and acetoacetyl-CoA. BPn could then, rely on its host cell for the supply in CoA as all other sequenced *Buchnera* strains [39], or rely on wPn.

5. Citric acid cycle

As expected, the complete citric acid cycle is missing in BPn, except alpha ketoglutarate dehydrogenase, which produce succinyl CoA, needed in lysine synthesis [39]. The loss of the citric acid cycle could be compensated by the fact that the bacterium has retained phosphotransacetylase (EC 2.3.1.8) and acetate kinase (EC 2.7.2.1) and is then able to generate ATP by the production of acetate from acetyl-CoA as an additional energy supply.

In particular, alpha ketoglutarate could be supplied to BPn by wPn which possess the gene coding for glutamate dehydrogenase which can generate alpha ketoglutarate by deamination of glutamate. This hypothesis is interesting as the source of alpha ketoglutarate was not known until today for any *Buchnera* species [39].

In wPn, we have detected nine genes implicated in the cycle, which is not surprising as other *Wolbachia* species like wMel have shown having retained the whole pathway [26] (Fig. 4f).

6. Protection against oxidative stress and iron toxicity

It has been demonstrated that *Wolbachia* can alter iron homeostasis in its host, reducing iron toxicity [40]. The required gene, bacterioferritin, is also present in wPn, suggesting this bacterium could provide protection to its host against oxidative stress [22].

It has also been showed that, in cases of stable associations, the presence of *Wolbachia* increases production by its host of reactive oxygen species and its antioxidant system [41], in a way that is positive for the host. Banana plant sap is rich in flavonoid compounds [42], shown to have negative and pro-oxidative effects on insects [43]. The effect of *Wolbachia* on the antioxidant system of the banana aphid could be essential for its survival on a flavonoid-rich diet. wPn also codes for a gluthatione transferase (EC 2.5.1.18) which also has antioxidant properties [44].

## Conclusions

Two bacterial endosymbionts are present in the banana aphid: *B. aphidicola* and *Wolbachia* sp. The presence of *B. aphidicola* was expected, but the systematic occurrence of *Wolbachia* raised question about the reason of its presence in *P. nigronervosa* and its importance for the aphid.

A symbiosis is considered to be obligate for both partners when the bacterium cannot be cultivated outside of the host and when eliminating the bacterium from the host could have severe consequences for its survival or reproduction. We cannot conclude about the possibility of culturing wPn but several microinjection tests made with antibiotics known to have a negative effect on *Wolbachia* in the literature [45] have led to the death of all the aphids tested.

In addition, our study suggests that BPn and wPn could complement each other in several important pathways. Both endosymbionts show functional complementarity in the synthesis of essential nutrients for their host. The most striking cases are the loss of lysine and riboflavin production in BPn, nutrients which are essential for the host and not known to be present in their diet. *Buchnera* is a symbiont in constant evolution and the loss of certain pathways could have been propelled by metabolic redundancies which have been eliminated through a long-term stable association with wPn. In addition, wPn also seems to be able to supply amino acid precursors to *Buchnera* and could play an important role in the activation of the antioxidant system of its host. Moreover, both symbionts are localized in the bacteriocytes, which can facilitate biological interactions between them.

All those information led us to hypothesize that wPn could, in the same way as BPn, be essential for the survival of its aphid host. In the last few years, more and more examples of co-obligate symbiosis have been discovered in insects [29, 46–48]. The acquisition of secondary symbionts that can replace primary symbiont functions seems to be a more common mechanism than expected, which could intensify the process of irreversible reduction of endosymbiont genomes [49].

More interestingly, several authors studying the aphids *Cinara cedri* and *C. tujafilina* have suggested, using a similar high-throughput sequencing methodology as ours, that the secondary symbiont *Serratia symbiotica* has evolved to form a deep and co-obligate association with *B. aphidicola* [37, 50]. This relation is based on the production and supply of essential vitamins and amino acids to the host with *Buchnera* having lost the ability to synthetize them.

For the first time, a similar evolution toward an obligatory symbiont is suggested for *Wolbachia* in aphids.

Nevertheless, all these hypotheses are based on the analysis of wPn and BPn draft genomes and should be completed by the study of other *P. nigronervosa* populations. Some questions remain about the expression of the genes detected in *Wolbachia*, as the quantity of the latter bacteria seems lower than the one of *Buchnera* in the bacteriocytes. Moreover, wPn was also detected in the insect body, outside of the bacteriocytes, which raises question about how the metabolites could be shared with BPn. Further, transcriptomic and metabolic studies and the complete genomes of both bacteria are needed to answer those interrogations.

This study however provides useful data about the relations between the banana aphid and its symbionts, a model that was little studied, despite its importance on banana production. These information about the vector of the BBTV could help in the future development and implementation of control methods of this disease, which is still a threat for bananas worldwide.

## Methods

### Insect samples

*Pentalonia nigronervosa* strains were collected at Madagascar (Ambodiafonsty), Burundi (Bujumbura), Gabon (Libreville), Rwanda (Kigali), Brazil (Piracicaba), and Australia (Tinbeerwah and Brisbane). Each strain was maintained in isolated growth chambers at 25 °C in a long-day photoperiod (16L8D) on banana plantlets of the Williams variety. Plants were replaced when they outgrew the boxes or when they were covered with honeydew.

### Aphid’s total DNA extraction

Adult aphids were soaked in 100 % ethanol for 1 min prior to total DNA extraction using the Qiagen DNeasy blood and tissue kit (QIAGEN, Chatworth, CA, USA). DNA quality was evaluated using a Nanodrop spectrophotometer (NanoDrop® ND-1000 Spectrophotometer, NanoDrop Technologies, Wilmington, DE, USA). Two ratios of absorbance were measured: 260/280 nm and 260/230 nm. DNA was considered to be of good quality and purity when those ratios were around 1.8 for 260/280 and in the range of 2.0–2.2 for 260/230. The DNA extraction was repeated twice on each strain.

### Diagnostic PCR detection of *Wolbachia* and *Buchnera* in each strain

The partial 16S ribosomal DNA (rDNA) sequences of the symbionts were amplified for each aphid strain by polymerase chain reaction (PCR) using the forward primer 16SA1_nt1 (5′-AGAGTTTGATCMTGGCTCAG-3′) in combination with reverse primer W2_nt435 (5′-CTTCTGTGAGTACCGTCATTATC-3′) for *Wolbachia*, or reverse primer ApisP1_nt298 (5′-TTCCAGTGTGGCTGGTTA-3′) for *Buchnera*. PCR temperature profile and the protocol were used as described [2]. Five individuals were tested for each strain and the amplification was repeated twice for each sample to confirm the result.

### Comparison between *Wolbachia* strains from hosts of different origins

To evaluate more in depth the diversity between the strains from Gabon, Madagascar, and Burundi, four important MLST genes of *Wolbachia* (GroEl, CoxA, GatB, and FtsZ) were amplified by PCR using the primers listed in Additional file 1 and the same PCR conditions as for the 16S rDNA amplification. The obtained sequences were sent for Sanger sequencing to Macrogen Europe and were compared through an alignment made with Muscle [51].

### In situ hybridization

*Buchnera* and *Wolbachia* in dissected embryos and bacteriome of the strain Madagascar were visualized by whole-mount fluorescence in situ hybridization as described [52], using the fluorochrome-labeled oligonucleotide probes listed in Additional file 5. AlexaFluor 555-labeled ApisP2 probe was used for *Buchnera* detection. For *Wolbachia* detection, we simultaneously used 43 fluorescein-labeled probes (Stellaris RNA probe, Biosearch Technologies) at a time because the fluorescence signals by standard method using one probe were too weak to be detected. Designing probes were conducted by using Stellaris Probe Designer (https://www.biosearchtech.com/stellarisdesigner/, Biosearch Technologies) with the specific gene sequence regions for 16S rRNA gene of *Wolbachia*. Then specificities of the probes were checked using ProbeCheck (http://131.130.66.200/cgi-bin/probecheck/probecheck.pl). Host cell nuclei were counterstained with 4′,6-diamidino-2-phenylindole. Observations were made using a laser scanning confocal microscope (LSM 5 Pascal; Carl Zeiss). The specificity of in situ hybridization was confirmed by the following control experiments: a no-probe control, an RNase digestion control as described [53].

### Selective elimination of *Wolbachia*

Following the method developed in [54], adult aphids from Madagascar, previously anesthetized with acetone [55], were injected with 0.1 μl of antibiotic solutions (10 mg/ml) per mg of body weight at the basement of a mid- or hind-leg using a fine glass needle. Two different antibiotics known to have selective elimination effect on secondary aphid symbionts [56] were tested: ampicillin and cefotaxime. In addition, tetracycline, an antibiotic known to eliminate *Wolbachia*, was tested [45]. This last antibiotic is although also known to have some deleterious effect on *Buchnera* [57].

Thirty adult aphids (G0) were injected with each antibiotic. For a control treatment, adult aphids were injected with filtered distilled water instead of the antibiotic solution.

The injected insects were reared individually to establish isofemale lines, and the nymphs born between 48 and 60 h after injection were collected. These nymphs were defined as G1 of each isofemale line. When G1 females became adult and produced a sufficient number of G2 offspring, G1 were collected and subjected individually to diagnostic PCR detection as described above to check for the presence of *B. aphidicola* and *Wolbachia*.

When G2 nymphs were viable, they were reared in the same manner to obtain G3 offspring and tested with the same PCR conditions as G1, and so on for G3 nymphs.

### Hemolymph and bacteriocytes collection and DNA extraction

Aphid strains, coming from Madagascar, Burundi, and Gabon were subjected to extraction of hemolymph-containing fragmented bacteriocytes.

Aphids were anesthetized using acetone then placed in a droplet of mineral oil to facilitate the collection. Hemolymph of one single adult aphid from each of the three strains was collected using a microinjection device (Nanoliter 2000 & SYS-Micro4 Controller, WPI USA) and subjected to whole genome amplification using the GenomePlex® Single Cell Whole Genome Amplification Kit (Sigma-aldrich, USA) according to the manufacturer’s instructions.

### High-throughput sequencing and bioinformatics analysis

The sequencing library was prepared using the Truseq DNA Sample Prep kit (Illumina, USA) and sequenced on HiSeq2000 (Illumina, USA) using the TruSeq PE Cluster Kit v3 and the Truseq SBS kit v3 (Illumina, USA). The software CLC Bio was used for de novo assembly of the reads using standard parameters (insertion cost = 3, deletion cost = 3, mismatch cost = 2, length fraction = 0.5, and similarity fraction = 0.8). Reads from each individual strain were mapped individually against the assembled contigs using CLC Bio software with the same standard parameters as for de novo assembly. The assembled contigs were also blasted against the prokaryotic 16S ribosomal RNA database of NCBI [58] using blastn with cut-off parameters of a E value <1 × 10^−5^ and a sequence length hit >50 nt.

The assembled contigs were further translated in the 6 open reading frames and blasted (blastx) against refseq protein database release 61 from NCBI using CLC Bio and the same cut-off parameters. Dedicated R [59] scripts were used to select subsets of contigs, for example, associated with a given genus or species, from the BLASTx output and then to create a fasta file containing the sequences of the selected contigs. The fasta file was processed on myRAST program [60] using standard parameters. This program annotated any gene present in the sequences and delivered a fasta file of the protein sequences associated to the gene calling. Finally, KAAS-KEGG Automatic Annotation Server [61] was used to assign KEGG Orthologs to the proteins. The protein fasta files were uploaded on the genome partial KAAS job request.

### Confirmation of gene pathways by PCR

Primers (Additional file 1) were designed to confirm the most interesting hypotheses gained from NGS data analyses. Genes that were tested are marked with a star in Fig. 4 (a) to (f). To confirm the absence in BPn of genes that were found in wPn genome, primers able to amplify the targeted genes in all the sequenced *Buchnera* strains were designed. In addition, primers specific to wPn were designed to confirm the presence in wPn of those genes.

In the same way, primers able to amplify the targeted genes in all the sequenced *Wolbachia* strains were designed to confirm the absence in wPn of genes that were found in the BPn genome. For each test, an *Acyrthosiphon pisum* was used as a positive control for *Buchnera.* For each primer pair, PCR amplifications were performed in 50-μl reactions containing 2 μl of DNA, 5 μl of 10× reaction buffer (Roche), 5 μl of dNTP (2.5 mM each), 1.5 μl of MgCl_2_ (50 mM), 1 μl of each primer (25 μM), 0.2 μl of Taq polymerase (Roche, 1 U/μl), and 34.3 μl of water. The temperature cycle corresponded to an initial denaturation step at 95 °C followed by 40 cycles of 30 s at 95 °C, 1 min at 52 °C, and 2 min at 72 °C.

When a gene was amplified, the obtained sequence was sent for Sanger sequencing to Macrogen Europe for confirmation.

### Data deposition

Sequence data has been deposited at NCBI under Bioproject PRJNA268300.The lists of wPn and BPn annotated genes can be found in Additional files 6 and 7 respectively.

## Additional files

Additional file 1: Table S1. List of primers used in this study. B: primers targeting *Buchnera* genus; W: primers targeting *Wolbachia* genus; wPn: specific primers for wPn. (XLSX 13 kb) Additional file 2: Table S2. 16s rRNA gene sequences analysis. (XLSX 15.9 kb) Additional file 3: Table S3. General features of *Wolbachia*’s genomes. (XLSX 10.7 kb) Additional file 4: Table S4. General features of *Buchnera*’s genomes. (XLSX 10.7 kb) Additional file 5: Table S5. In situ hybridization probes used in this study. (XLSX 11.7 kb) Additional file 6: Table S6. List of wPn annotated genes. (XLSX 31.4 kb) Additional file 7: Table S7. List of BPn annotated genes. (XLSX 19.5 kb)
